# Cyclooxygenase 2 (COX2) and Peroxisome Proliferator-Activated Receptor Gamma (PPARG) Are Stage-Dependent Prognostic Markers of Malignant Melanoma

**DOI:** 10.1155/2010/848645

**Published:** 2009-07-20

**Authors:** Stefanie Meyer, Thomas Vogt, Michael Landthaler, Anna Berand, Albrecht Reichle, Frauke Bataille, Andreas H. Marx, Anne Menz, Arndt Hartmann, Leoni A. Kunz-Schughart, Peter J. Wild

**Affiliations:** ^1^Department of Dermatology, University of Regensburg, 93042 Regensburg, Germany; ^2^Department of Hematology and Oncology, University of Regensburg, 93042 Regensburg, Germany; ^3^Institute of Pathology, University of Regensburg, 93042 Regensburg, Germany; ^4^Institute of Pathology, University Medical Center Hamburg-Eppendorf, 20251 Hamburg, Germany; ^5^Institute of Clinical Pathology, University of Erlangen, 91054 Erlangen, Germany; ^6^OncoRay - Center for Radiation Research in Oncology, TU Dresden, 01307 Dresden, Germany; ^7^Institute of Surgical Pathology, University Hospital Zurich, Schmelzbergstrasse 12, 8091 Zurich, Switzerland

## Abstract

Using tissue microarrays (TMAs) we studied COX2/PPARG immunoreactivity in a broad spectrum of tumors focussing on clinicopathological correlations and the outcome of patients with malignant melanoma (MM). TMA-1 contained normal and tumor tissues (*n* = 3448) from 47 organs including skin neoplasms (*n* = 323); TMA-2 88 primary MM, 101 metastases, and 161 benign nevi. Based on a biomodulatory approach combining COX/PPAR-targeting with metronomic low-dose chemotherapy metastases of 36 patients participating in a randomized trial with metastatic (stage IV) melanoma were investigated using TMA-3. COX2/PPARG immunoreactivity significantly increased from nevi to primary MM and metastases; COX2 positivity was associated with advanced Clark levels and shorter recurrence-free survival. Patients with PPARG-positive metastases and biomodulatory metronomic chemotherapy alone or combined with COX2/PPARG-targeting showed a significantly prolonged progression-free survival. Regarding primary MM, COX2 expression indicates an increased risk of tumor recurrence. In metastatic MM, PPARG expression may be a predicitive marker for response to biomodulatory stroma-targeted therapy.

## 1. Introduction

Cyclooxygenases (COXs) catalyze the first rate-limiting step in the conversion of arachidonic acid to prostaglandins. Two COX isoenzymes have been identified: COX1 is constitutively expressed in most tissues and mediates the synthesis of prostaglandins in normal physiological processes, whereas COX2 is not detectable in most normal tissues but is rapidly induced by various stimuli such as inflammatory reactions [1]. COX2 is also expressed in various tumor types [2], and levels of expression have been shown to correlate with invasiveness and prognosis in some tumor entities, suggesting an important role of COX2 in tumor development and progression. Epidemiological studies show that prolonged COX2 inhibition through acetylsalicylic acid or other nonsteroidal anti-inflammatory drugs (NSAIDs) might offer some protection against colon cancer and some other malignancies [3, 4]. Accordingly, in animal experiments COX2 inhibitors can reduce the incidence of colon carcinoma in APC knockout mice treated with chemical carcinogens [5]. The mechanism by which COX2 expression accelerates tumorigenesis is poorly understood. However, a potential role of COX2 in epithelial and melanocytic skin cancer development is also not unlikely, since COX2 is frequently expressed in malignant melanomas (MMs) [6, 7] and squamous cell carcinomas of the skin [8, 9]. 

The peroxisome proliferator-activated receptor (PPAR) is a member of the nuclear hormone receptor subfamily of ligand-activated transcription factors. There are three known subtypes of peroxisome proliferator-activated receptors; PPARA, PPARD, and PPARG. The latter is involved in physiological adipocyte differentiation and differentially expressed in several types of human cancers [10], for example, in prostate cancer [11, 12], breast adenocarcinomas [13], overian cancer [14, 15], lung cancer [16], and colon cancer [17]. Accordingly, PPAR ligands were shown to inhibit the growth of cells from different cancer lineages in vitro [18]. In human melanoma cell lines the antiproliferative and apoptosis-inducing effect of PPARG ligands was demonstrated, too [19, 20]. 

Current research data and clinical experience suggest that PPARA/G can mediate both direct antitumoral and immunomodulatory effects and a broad spectrum of stroma modulating activity including antiangiogenic, anti-inflammatory, and immunoaugmentative effects [21, 22]. Examples of superadditive complementation of PPARG agonists by COX2 inhibitors and metronomic chemotherapy are well-documented experimentally and in clinical trials, respectively [10, 16, 23]. 

We had studied such combined tumor-stroma-targeted cancer therapy using PPARG agonists and COX2 inhibitors in the second-line treatment of advanced metastatic melanoma disease [22, 23]. In a randomized multi-institutional phase II trial including 76 mostly chemorefractory patients with progression of metastatic melanoma (stage IV melanoma according to AJCC criteria), we had observed a significantly prolonged progression-free survival in the group of patients that received angiostatically scheduled low-dose metronomic chemotherapy (trofosfamide) in combination with a PPARG agonist (pioglitazone) and a COX2 inhibitor (rofecoxib) compared to the group of patients who received metronomic chemotherapy alone [22]. Accordingly, tumor-associated inflammatory and angiogenic processes mediated by COX2 overexpression or PPARG deficiency were suggested to play a pivotal role in the biology of melanoma progression [22]. However, there is insufficient data on the expression of both target molecules; therefore, their prognostic and therapeutic relevance in MM is still unclear. 

The study presented herein is based on a high-throughput tissue microarray (TMA) analysis, a highly efficient technology for investigating large numbers of tumors. To the best of our knowledge this is the largest study of this topic which can link expression data with extensive follow-up data of melanoma patients, respectively. In addition, as we gather extensive data on various other cancers and normal tissues (47 organs and tissue entities) we can put the specifities of the melanoma data into a broader oncologic context.

## 2. Materials and Methods

### 2.1. Tissue Microarrays (TMAs)

TMA construction was performed as described previously [24]. The local Institutional Review Boards of the Universities of Regensburg and Basel granted approval for this project. 

The first TMA (*TMA-1*) contained formalin-fixed, paraffin-embedded tissue punches from the archives of the Institute of Pathology, University of Basel, Switzerland. A comprehensive TMA was created by transferring representative tissue cylinders with a diameter of 0.6 mm to seven new paraffin blocks as described by Bubendorf et al. [25]. Representative areas of different subtypes for the most frequent tumor entities and their corresponding nontumorous tissue were selected for analysis. Four *μ*m sections of the resulting TMA block were cut and mounted to an adhesive-coated slide system (Instrumedics Inc. Hackensack, NJ, USA). The constructed multitumor TMA-1 consisted of 3448 primary tumors from 132 different tumor subtypes and 26 different normal tissues and allowed us to determine the prevalence of COX2 and PPARG expression in nontumorous tissues and corresponding malignant tumors. Samples from skin (*n* = 330), lung (*n* = 217), brain (*n* = 228), breast (*n* = 218), colon (*n* = 204), soft tissue (*n* = 150), salivary gland (*n* = 152), testis (*n* = 126), ovary (*n* = 140), and kidney (*n* = 144) were the major tissues assembled on this TMA. The evaluation of tissue and clinical data was performed on the basis of anonymized patient data according to the regulations of the University of Basel Institutional Review Board. Detailed tumor and tissue characteristics can be found in supplementary Tables 1 and 2 in Supplementary Material available online at doi:10.1155/2010/848645. The skin-related data sets were extracted and are summarized in Table 1.

The second TMA (*TMA-2*) was constructed as described by Wild et al. [26] and contained a total of 350 formalin-fixed, paraffin-embedded human tissues: 88 (25.1%) primary malignant melanomas, 101 (28.9%) metastases, and 161 (46.0%) benign nevi. H&E-stained slides of all tumors were evaluated by two surgical pathologists (T.V., P.J.W.). Clinical follow-up data, provided by the Central Tumor Registry of the University of Regensburg, were available for all patients with primary malignant melanomas (*n* = 88). The median follow-up for all patients was 54 months (range 0 to 135 months), whereas the median follow-up for censored patients (*n* = 74) was 63.5 months. Characteristic parameters of TMA-2 are summarized in Table 2.

The third TMA (*TMA-3*) was constructed on the basis of a randomized multi-institutional phase II trial using an angiostatic biomodiulatory approach to assess the impact of COX2- and PPAR-targeted therapy in combination with metronomic low-dose chemotherapy in patients with advanced metastatic stage IV melanoma [22]. The clinical trial was designed to select metronomic chemotherapy alone (arm A: trofosfamide 50 mg orally three times daily, day 1+) or combined anti-inflammatory/angiostatic treatment (arm B: trofosfamide as mentioned above plus rofecoxib 25 mg orally, day 1+, and pioglitazone 60 mg orally, day 1+) for further evaluation. A total of 76 patients, mostly (>60%) refractory to at least one previous chemotherapy with maximum tolerated doses, and progression of metastatic melanoma were included; from the Institute of Pathology and the Department of Dermatology (University of Regensburg, Germany) 194 formalin-fixed paraffin-embedded metastatic tissues of 36 patients (47%) were available for further immunohistochemical analysis. The local ethic committee had approved the study.

Prior to TMA-construction, H&E-stained slides of all specimens were evaluated by two dermatopathologists (T.V., S.M.) to identify representative metastatic areas. Clinical follow-up data with a median follow-up period of 9 months (range 1–43 months) were available for 35 melanoma patients (97%), that is, 12 patients (33%) who received metronomic chemotherapy alone (arm A) and 23 patients (64%) with combined anti-inflammatory/angiostatic treatment (arm B). Median follow-up of censored patients was 7 months (range 2–43 months). Characteristic parameters of TMA-3 are given in Table 4.

### 2.2. Immunohistochemistry (IHC)

Immunohistochemical studies utilized an avidin-biotin peroxidase method with a 3-amino-9-ethylcarbazole (AEC) chromatogen. After antigen retrieval (steam boiler with citrate-buffer, pH 6.0 for 20 minutes) immunohistochemistry was carried out applying the ZytoChemPlus HRP Broad Spectrum Kit (Zytomed Systems, Berlin, Germany) according to the manufacturer's instructions. The following primary antibodies were used: anti-COX2 (mouse monoclonal, Cayman Chemical, Ann Arbor, Mich, USA; dilution 1 : 200, final concentration 2.5 *μ*g/mL), anti-PPARG (rabbit monoclonal, Cell Signalling, New England Biolabs GmbH, Frankfurt am Main, Germany; dilution 1 : 400), anti-TP53 (mouse monoclonal IgG, clone Bp53-12 (sc-263), Santa Cruz Biotechnology Santa Cruz, Calif, USA; dilution 1 : 1000), and anti-Ki-67 (rabbit monoclonal, clone MIB1; DakoCytomation GmbH, Hamburg, Germany; dilution 1 : 10, final concentration 5 *μ*g/mL). As a positive control for COX2 and PPARG IHC, a colon carcinoma with known COX2 and PPARG expression was chosen. Normal tissue samples of 10 different organs were considered as negative controls. Two pathologists performed a blinded evaluation of the stained slides. Cytoplasmic COX2 and nuclear PPARG immunoreactivity were estimated using an arbitrary semiquantitative four-step scoring system (0-3+), based on the intensity of cytoplasmic COX2 staining [6] and the percentage of PPARG positive cell nuclei [7]: 0 (negative): no cytoplasmic COX2 staining/PPARG staining 0% of cell nuclei; 1+: weak COX2 staining/PPARG staining 1 to 9%; 2+: moderate COX2 staining/PPARG staining 10 to 50%; 3+: strong COX2 staining/ PPARG staining greater than 50%. Causes of noninterpretable results included lack of tumor tissue and presence of necrosis or crush artifact. The percentage of tumor cells with nuclear Ki-67 and TP53 staining was determined as described previously [27]. Ki-67/TP53 labeling was considered high if at least 5% of the tumor cells were positive.

### 2.3. Statistical Analysis

Specimens on TMA-1 and TMA-2 were considered independently. Concerning TMA-3, COX2 and PPARG immunoreactivity were examined for a mean of 5 metastatic samples per patient (range 1–15); the median level of COX2 and PPARG immunoreactivity was chosen for further analyses using the SPSS version 16.0 (SPSS, Chicago, Ill, USA). *P*-values <.05 were considered significant. Contingency table analysis and two-sided Fisher's exact tests or *X* ^2^-tests were used to study statistical associations between clinicopathological and immunohistochemical data. Retrospective overall and progression-free survival curves comparing patients with and without any of the variables were calculated using the Kaplan-Meier method, with significance evaluated by two-sided log rank statistics. For the analysis of progression-free survival, patients were censored at the time of their last progression-free clinical follow-up appointment. For the analysis of overall survival, patients were censored at the time of their last clinical follow-up appointment or at their date of death not related to the tumor. For multiple testing, the closed test principle was used.

## 3. Results

### 3.1. TMA-1

Investigation of COX2 and PPARG protein expression in 323 benign and malignant skin tumors using a comprehensive multitumor TMA (TMA-1) was informative in 57.6% (186/323) and 65.6% (212/323) of cases. COX2 and PPARG expression of any intensity (score 1+-3+) was detected in 81.7% (152/186) and 32.5% (69/212) of informative cases, respectively. Table 1 summarizes the expression data and statistical analysis of COX2 and PPARG immunoreactivity of each skin tumor entity on TMA-1. For connective tissue tumors (Kaposi sarcoma, capillary hemangioma, benign histiocytoma) no significant differences could be found in benign versus malignant tumors (*P* = .61 and *P* = .13). Regarding epithelial tumors (squamous cell carcinomas, basal cell carcinomas) positive PPARG staining was detected significantly more often in basal cell carcinomas than in squamous cell carcinomas (*P* = .001). Surprisingly, 86.9% of benign skin adnexal tumors (sebaceous adenomas) were positive for COX2; 21.7% positive for PPARG. Regarding melanocytic lesions, 100% (38/38) of primary melanomas and 78.9% (15/19) of benign nevi revealed at least weak COX2 immunoreactivity (score 1+-3+); 48.7% (20/41) of primary melanomas and 8.3% (2/24) of benign nevi demonstrated PPARG positivity (1+-2+). Accordingly, compared to benign nevi, expression of both COX2 and PPARG was significantly increased in primary melanomas (*P* = .02 and *P* = .001). 

Besides skin tumors, COX2 and PPARG expression was analyzed in many other benign and malignant tissue types from 46 different organs using a comprehensive multitumor TMA-1. As shown in supplementary Tables 1 and 2, differential COX2 and PPARG expression between normal and neoplastic tissue could be observed for almost every tissue type investigated. In prostate cancer, for example, COX2 expression continuously increased from prostatic hyperplasia to prostatic intraepithelial neoplasia (PIN) to organ-confined prostate cancer to hormone-refractory prostate cancer to metastatic disease (supplementary Figures 1A).

### 3.2. TMA-2

**Figure 1:**
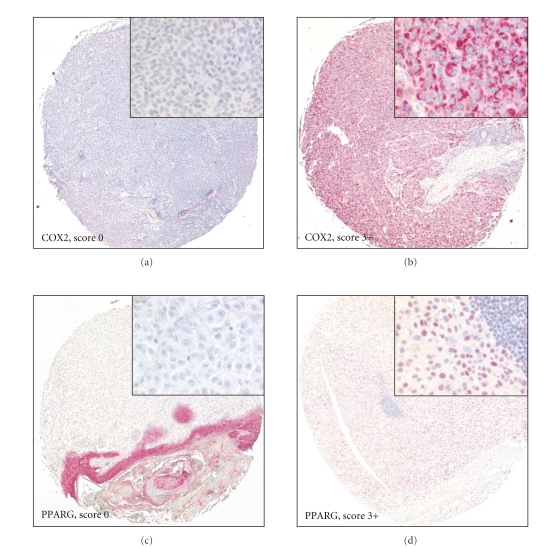
Immunohistochemical COX2 and PPARG staining of malignant melanomas on TMA-2. Original magnification 10x (insets 200x). Representative examples of a primary malignant melanoma with negative (a) and strong (b) immunoreactivity for COX2. Representative examples of a primary malignant melanoma with negative (c) and strong (d) immunoreactivity for PPARG.

**Figure 2:**
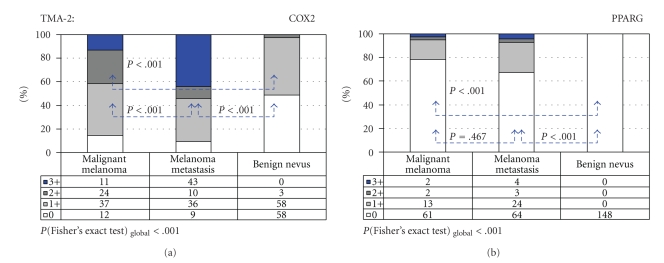
Cumulative bar charts of COX2 (a) and PPARG (b) immunoreactivity in melanocytic skin tumors using TMA-2.

Based on the results of TMA-1, a second TMA (TMA-2) with clinical follow-up data sampling primary malignant melanomas and melanoma metastases as well as benign nevi was constructed. COX2 and PPARG immunoreactivity was informative in 86.0% (301/350) and 91.7% (321/350) of cases, respectively. Expression of COX2 and PPARG of any intensity was detected in 73.8% (222/301) and in 15.0% (48/321) of informative cases. Representative negative and positive COX2 and PPARG immunostaining patterns in malignant melanoma are shown in Figures 1(a)–1(d). Figures 2(a)  and 2(b)  summarize the results of COX2 and PPARG IHC for primary melanomas, metastases, and nevi on TMA-2. The percentage of COX2 positive cases significantly increased from benign nevi (51%) to primary melanomas (86%) and melanoma metastases (91%; *P* < .001; Figure 2(a)). Likewise, PPARG immunoreactivity significantly increased from benign nevi (0%) to malignant melanomas (22%) and melanoma metastases (33%; *P* < .001; Figure 2(b)). Clinicopathologic variables of melanoma patients were correlated with COX2 and PPARG expression (Table 2). In primary melanomas, positive COX2 immunoreactivity was significantly related to advanced Clark levels (*P* = .004), but no other clinicopathologic variables such as tumor growth pattern, p53 immunoreactivity, and Ki-67 labeling index. Skin metastases demonstrated a gradually weaker COX2 immunoreactivity compared with lymph node metastases (*P* = .013). Among the various types of benign nevi on TMA-2, COX2 expression was significantly increased in congential nevi compared to compound, junctional, and dermal melanocytic nevi (*P* < .001). 

**Figure 3:**
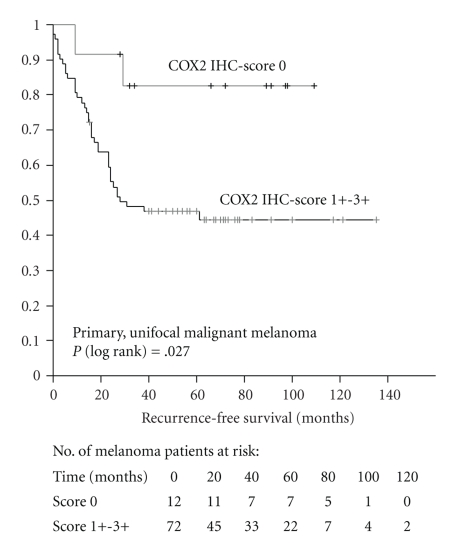
Distribution of time (months) to tumor-related death among patients with primary malignant melanomas showing negative (0) or positive (1+ to 3+) COX2 immunoreactivity as estimated by the Kaplan Meier method.

According to a univariate analysis, tumor progression was significantly related to both melanoma thickness and COX2 immunoreactivity, respectively (*P* = .03; Table 3); that is, expression of COX2 was associated with shorter progression-free survival (*P* = .03; Figure 3). In contrast, PPARG expression of primary melanomas was not associated with any of the variables neither the clinicopathologic ones nor progression-free and overall survival (Tables 2  and 3).

### 3.3. TMA-3

Using TMA-3, the prognostic and therapeutic meaning of COX2 and PPARG expression was analyzed in patients with advanced metastatic melanoma disease (*n* = 36). All patients received angiostatic biomodulatory treatment with trofosfamide alone (arm A, *n* = 12) or in combination with rofecoxib and pioglitazone (arm B, *n* = 24). COX2 and PPARG protein expression of metastatic tissues was informative in all 36 cases. Expression of COX2 and PPARG of any intensity was detected in 97.2% (35/36) and in 38.9% (14/36) of patients, respectively. Clinicopathologic variables of this cohort of patients with advanced metastatic melanoma disease were compared relative to COX2 and PPARG expression (Table 4). 

**Figure 4:**
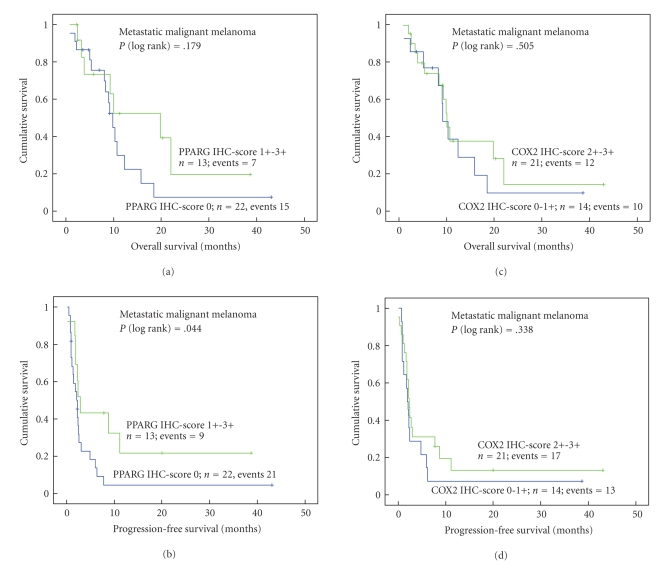
Distribution of time (months) to death and tumor progression among patients with advanced metastatic melanomas in correlation with immunoreactivity of PPARG (a), (b) or COX2 (c), (d). All patients received biomodulatory treatment. The calculation was performed according to the method of Kaplan and Meier.

Considering all 36 patients receiving biomodulatory therapy expression of PPARG (score 1+-3+) in the metastases was significantly associated with longer progression-free survival (*P* = .044) but not with overall survival (*P* = .179; Figures 4(a)  and 4(b)). Expression of COX2 (score 2+-3+) in the metastases, however, was not associated with overall and progression-free survival, respectively (Figures 4(c)  and 4(d)). Besides PPARG immunoreactivity, stage of the primary melanoma was also a significant prognostic factor for progression-free survival (*P* = .016; Table 4). In a multivariate Cox regression model, using primary tumor stage (pTis-pT3 versus pT4) and PPARG expression (negative versus positive) as covariates, neither PPARG immunoreactivity nor primary tumor stage remained significant (data not shown).

## 4. Discussion

In this study, we demonstrate by a comprehensive multitumor TMA that COX2 and PPARG are differentially expressed in a broad spectrum of normal and malignant tissues. Focussing on tumors of the skin we can further confirm that COX2 immunoreactivity of primary MM is significantly associated with advanced Clark levels (*P* = .004) and shorter recurrence-free survival (*P* = .03). PPARG expression of primary MM, however, does not provide significant prognostic information. Yet, by analysis of COX2 and PPARG expression in MM metastases of patients who had received biomodulatory therapy, we can show that only the expression of PPARG is significantly associated with longer progression-free survival (*P* = .044). These findings suggest that COX2 may mainly contribute to early steps in melanoma progression, that is, growth and invasion of primary MM, and becomes less essential in the advanced metastatic setting of melanoma disease. Our study confirms the prognostic meaning of COX2 in patients with primary MM and adds a new late-stage histolpathological marker, PPARG, which may be predictive for responsiveness to biomodulatory therapy in advanced metastatic MM. To our knowledge this is the first TMA study demonstrating that PPARG protein expression may be a positive prognostic marker indicating responsiveness to stroma-targeted therapy in the late metastatic stage (IV) of MM disease, that is, in patients refractory to conventional first-line chemotherapy, mostly with dacarbacine. 

Consistent with previously published data on melanocytic skin lesions [6, 7] our immunohistochemical analysis of benign nevi, primary MM and MM metastases show that COX2 and PPARG immunoreactivity significantly increases from benign nevi to primary MM and MM metastases. In other organs, however, for example, in primary cancers of the lung versus normal lung tissues, decreased expression levels of PPARG were found and associated with poor prognosis [16]. At first sight, these findings are in contrast to the upregulation of PPARG in primary MM and MM metastases versus benign nevi observed with TMA-2. But, as our data also show, this upregulation does not correlate with the outcome of MM patients indicating a distinct role of PPARG in primary MM and MM metastases. Notably, in the advanced metastatic stages of MM enclosed in this study, patients with PPARG-positive metastases versus PPARG-negative metastases show a significant survival benefit concerning progression-free survival (*P* = .044) *not* dependent on whether angiostatically scheduled metronomic chemotherapy (trofosfamide) was administered alone or in combination with pioglitazone (PPARG agonist) and rofecoxib (COX2 inhibitor) as additional biomodulatory therapy. Considering PPARG or COX2 as candidate substrates for targeted cancer therapy, it could be assumed that only patients with PPARG- or COX2-positive metastases and additional PPARG-agonistic or COX2-inhibitory therapy would show a survival benefit compared with patients treated with metronomic chemotherapy alone. Yet, subgroup analysis with TMA-3 did not show a significant survival benefit for these patients. Thus, our study supports current concepts that targeting COX2 and PPAR is more a tumor-stroma effective approach than an approach depending on the status of target expression of the tumor itself [21, 22]. Possible explanations of this paradoxon are multifaceted and complex. There may be numerous “off-target” effects of the involved drugs, for example, modulation of COX2/PPARG-independent pathways [16, 18, 21]. According to the paradigm of biomodulatory stroma targeting approaches [21, 28] the effects may be indirect due to modifying the tumor stroma; that is, the therapy mainly exploits the dependence of cancer tissues on functions of the stroma providing a permissive and supportive environment for tumor cell survival, growth, invasion, and formation of metastases. A variety of soluble agents such as chemokines, growth factors, lipids, angiogenetic factors, proteinases, and proteinase inhibitors are involved in a complex crosstalk between tumor and stroma. Stroma targeted approaches aim to inhibit tumor growth and invasion by disruption of this tumor-stroma interaction. Interestingly, stromal cells in the tumoral microenvironment can also differ from their normal counterparts in the expression of biologically meaningful molecules [29] including also COX2 and PPARG expression. For instance, upregulation of these effectors could be detected in stromal myofibroblasts surrounding colon adenocarcinomas [30]. 

Therefore, to fully evaluate and understand the potential of COX2 and PPAR modulation in MM further studies using TMAs punching the surrounding stroma may be interesting future work. Based on the large comprehensive amount of data gained in this study it seems to be promising to further develop experimental protocols that employ COX2/PPAR biomodulation. The combination of both drugs is a logical consequence of experimental studies indicating that COX2 and PPARG signalling pathways are multiply intertwined: PPARG ligands suppress COX2 expression induced by lipopolysaccharide and phorbol myristate acetate in macrophages, astrocytes, and epithelial cells [16]. Moreover, expression of COX2 was suggested to be regulated by a negative feedback loop involving PPARG and NF-*κ*B [31, 32]. PPARG agonists were shown to downregulate COX2, potentiate the apoptotic effects of chemotherapeutic agents, and inhibit the growth of human melanoma cell lines in vitro [19, 20]. Consistently, the randomized phase II trial by Reichle et al. [22] including chemorefractory patients with progressive metastatic stage IV melanoma disease demonstrated a significantly prolonged progression-free survival if metronomic low-dose chemotherapy (trofosfamide) was combined with pioglitazone (PPARA and G agonist) and rofecoxib (COX2 inhibitor). In summary, COX inhibitors and PPAR agonists are a beneficial adjunct in biomodulatory therapy of MM rather independent of the presence of the targeted substrates in the cancer cells themselves. The expression of PPARG in the cancer, however, can indicate a higher probability to respond to stroma-targeted approaches also without drugs aiming on PPAR.

In conclusion, our study provides a late-stage prognostic marker, PPARG expression, which correlates with responsiveness to biomodulatory stroma-targeted therapy. But it should be kept in mind that the indication for such approaches cannot be solely based on selected features of the cancer cell itself but must consider the complexitiy of the stroma-tumor interaction, that is, the microenvironment, including angiogenesis, immunoeffects, and functions of the connective tissue as well. Therefore, further prospective clinical trials are needed to validate the meaning of PPARG and COX2 targeting as a part of biomodulatory therapeutic approaches.

## Figures and Tables

**Table 1:** COX2 and PPARG expression analysis of skin tumors using TMA-1.

Tumor entity	Cytoplasmic COX2 immunoreactivity						Nuclear PPARG immunoreactivity						*P* ^†^
	*n* analyzable	0 (*n*)	1+ (*n*)	2+ (*n*)	3+ (*n*)	P*	*n* analyzable	0 (*n*)	1+ (*n*)	2+ (*n*)	3+ (*n*)	P*	

*TMA-1:* total (*n* = 323)	186	34	86	54	12		212	143	50	19	0		**.0003**
Melanocytic lesions													
* *Malignant melanoma	38	0	16	17	5	**.02**	41	21	8	12	0	**.001**	**.01**
* *Benign nevus	19	4	7	8	0		24	22	2	0	0		1.00
Epithelial tumors													
* *Squamous cell carcinoma	30	3	10	11	6	.07	33	23	7	3	0	**.001**	.62
* *Basal cell carcinoma	31	7	16	7	1		33	11	21	1	0		.57
Connective tissue tumors													
* *Kaposi sarcoma	15	6	8	1	0	.61	18	13	5	0	0	.13	1.00
* *Benign histiocytoma	16	8	7	1	0		22	19	1	2	0		.47
* *Capillary hemangioma	14	3	9	2	0		18	16	2	0	0		1.00
Appendix tumors													
* *Benign sebaceous adenoma	23	3	13	7	0		23	18	4	1	0		1.00

*Fisher's exact test (2-sided); bold face representing significant data. ^†^Fisher's exact test (2-sided); association of COX2 and PPARG IHC within single tumor entities.

**Table 2:** Clinicopathologic parameters in relation to COX2 immunohistochemistry using TMA-2.

Variable * *Categorization	Cytoplasmic COX2 immunoreactivity						Nuclear PPARG immunoreactivity					*P* ^†^
	*n* analyzable	0 (*n*)	1+ (*n*)	2+ (*n*)	3+ (*n*)	*P* ^†^	*n* analyzable	0 (*n*)	1+ (*n*)	2+ (*n*)	3+ (n)	

Primary malignant melanomas												
* *Clark level												
* * * * * * * * * *II	4	3	1	0	0	**.004**	2	2	0	0	0	.793
* * * * * * * * * *III	14	3	6	3	2		12	11	1	0	0	
* * * * * * * * * *IV	52	2	27	15	8		52	39	10	2	1	
* * * * * * * * * *V	13	4	2	6	1		11	9	1	0	1	
* *Tumor thickness												
* * * * * * * * * * ≤2.0 mm	35	8	17	6	4	.104	31	24	6	1	0	.762
* * * * * * * * * * >2.0 mm	49	4	20	18	7		47	37	7	1	2	
* *Growth pattern*												
* * * * * * * * * *SSM	37	6	15	11	5	.748	8	5	3	0	0	.685
* * * * * * * * * *LMM	3	2	0	1	0		36	29	6	1	0	
* * * * * * * * * *NM	29	2	14	9	4		5	4	1	0	0	
* * * * * * * * * *ALM	6	1	3	1	1		27	21	3	1	2	
* * * * * * * * * *ONA	9	1	5	2	1		2	2	0	0	0	
* *TP53 immunoreactivity												
* * * * * * * * * * <5%	67	11	28	20	8	.308	63	49	10	2	2	.883
* * * * * * * * * * ≥5%	15	0	8	4	3		15	12	3	0	0	
* *Ki-67 labeling index												
* * * * * * * * * * <5%	68	11	29	18	10	.295	64	53	9	1	1	.101
* * * * * * * * * * ≥5%	14	0	7	6	1		14	8	4	1	1	
Melanoma metastases												
* *Lymph node	42	3	9	4	26	**.013**	42	32	6	2	2	.136
* *Skin	56	6	27	6	17		53	32	18	1	2	
Benign nevi												
* *Compound & junctional	47	39	7	1	0	<**.001**	53	53	0	0	0	—
* *Dermal	21	15	6	0	0		45	45	0	0	0	
* *Congenital	51	4	45	2	0		50	50	0	0	0	

*SSM, superfical spreading melanoma; LMM, lentigo maligna melanoma; NM, nodular melanoma; ALM, akro-lentiginous melanoma; NOS, not otherwise specified. ^†^Fisher's exact test (two-sided), bold face representing significant data.

**Table 3:** Univariate analysis of factors regarding tumor progression and death.

Variable * *Categorization	Tumor progression			Death		
	*n* ^a^	events	*P* ^b^	*n* ^a^	events	*P* ^b^

Age at diagnosis						
* * * * * * * * * * ≤60 years	48	25	0.7	48	7	0.6
* * * * * * * * * * >60 years	40	18		40	7	
Gender						
* * * * * * * * * *female	39	15	0.06	39	5	0.4
* * * * * * * * * *male	49	28		49	9	
Clark level^(c)^						
* * * * * * * * * *II	5	0	0.4	5	0	0.3
* * * * * * * * * *III	15	8		15	2	
* * * * * * * * * *IV	54	28		54	8	
* * * * * * * * * *V	13	7		13	4	
Tumor thickness						
* * * * * * * * * * ≤2.0 mm	38	14	**0.03**	38	4	0.2
* * * * * * * * * * >2.0 mm	50	29		50	10	
Ki67 labeling index						
* * * * * * * * * * <5%	33	17	0.7	33	7	0.9
* * * * * * * * * * ≥5%	36	16		36	7	
Cytoplasmic COX2 IHC						
* * * * * * * * * *score 0	12	2	**0.03**	12	0	0.1
* * * * * * * * * *score 1+-3+	72	39		72	14	
Nuclear PPARG IHC						
* * * * * * * * * *score 0	61	28	0.2	61	11	0.6
* * * * * * * * * *score 1+-3+	17	10		17	2	

^a^Only initial and unifocal malignant melanomas were included; ^b^Log rank test (two-sided), bold face representing significant data; ^c^According to UICC: TNM Classification of Malignant Tumours. 6th edn (2002) Sobin LH, Wittekind CH (eds.) Wiley, New York.

**Table 4:** Univariate analysis of factors regarding tumor progression and death using TMA-3.

Variable * * Categorization	Death			Tumor progression		
	*n*	events	P*	*n*	events	P*

Advance melanoma patients						
* *Age						
* * * * * * * * * * <60 years	12	7	0.152	12	11	0.163
* * * * * * * * * * ≥60 years	22	14		22	18	
* *Initial tumor stage						
* * * * * * * * * *pT1	2	1	0.690	2	2	**0.016**
* * * * * * * * * *pT2	1	1		1	1	
* * * * * * * * * *pT3	13	9		13	11	
* * * * * * * * * *pT4	9	6		9	6	
* * * * * * * * * *Melanoma in situ	1	0		1	1	
* *Initial regional lymph node status						
* * * * * * * * * *pN0	11	6	0.980	11	9	0.894
* * * * * * * * * *pN1	9	8		9	8	
* * * * * * * * * *pN2	6	4		6	5	
* * * * * * * * * *pN3	2	1		2	1	
* *Study therapy						
* * * * * * * * * *A: trofosfamide	12	10	0.570	12	10	0.898
* * * * * * * * * *B: trofosfamide + rofecoxib + pioglitazone	23	12		23	20	
* *CRP						
* * * * * * * * * *normal or < 30% decrease or increase	14	9	0.115	14	11	0.128
* * * * * * * * * * > 30% decrease	10	10		10	10	
* *Cytoplasmic COX2 IHC						
* * * * * * * * * *score 0 to 1+	14	10	0.505	14	13	0.338
* * * * * * * * * *score 2+ to 3+	21	12		21	17	
* *Nuclear PPARG IHC						
* * * * * * * * * *score 0	22	15	0.179	22	21	**0.044**
* * * * * * * * * *score 1+ to 3+	13	7		13	9	

*Log-rank test (two-sided).

## Abbreviations

MM: Malignant melanoma
TMA: Tissue microarray
IHC: Immunohistochemistry
COX2: Cyclooxygenase 2
PPARG: Peroxisome proliferator-activated receptor gamma
